# A narrow microbeam is more effective for tumor growth suppression than a wide microbeam: an *in vivo* study using implanted human glioma cells

**DOI:** 10.1107/S090904951101185X

**Published:** 2011-05-17

**Authors:** Atsushi Uyama, Takeshi Kondoh, Nobuteru Nariyama, Keiji Umetani, Manabu Fukumoto, Kunio Shinohara, Eiji Kohmura

**Affiliations:** a Department of Neurosurgery, Kobe University Graduate School of Medicine, Kobe, Hyogo 650-0017, Japan; b Japan Synchrotron Radiation Research Institute, Hyogo, Japan; c Institute of Development, Aging and Cancer, Tohoku University, Sendai, Japan; d Advanced Research Institute for Science and Engineering, Waseda University, Tokyo, Japan

**Keywords:** microbeam radiation therapy, narrow microbeam, wide microbeam, co-planar microbeam, cross-planar microbeam

## Abstract

The tumoricidal mechanisms of microbeam radiation therapy, and the more recently proposed minibeam radiation therapy, for the treatment of brain tumors are as yet unclear. Moreover, from among the various parameters of beam geometry the impact of changing the beam width is unknown. In this study, suppression of tumor growth in human glioma cells implanted in mice was evaluated experimentally using microbeams of two different widths: a conventional narrow beam (20 µm width, 100 µm center-to-center distance) and a wide beam (100 µm width, 500 µm center-to-center distance). The tumor growth ratio was compared and acute cell death was studied histologically. With cross-planar irradiation, tumor growth was significantly suppressed between days 4 and 28 after 20 µm microbeam irradiation, whereas tumor growth was suppressed, and not significantly so, only between days 4 and 18 after 100 µm microbeam irradiation. Immunohistochemistry using TUNEL staining showed no increase in TUNEL-positive cells with either microbeam at 24 and 72 h post-irradiation. The 20 µm microbeam was found to be more tumoricidal than the 100 µm microbeam, and the effect was not related to apoptotic cell death. The underlying mechanism may be functional tissue deterioration rather than direct cellular damage in the beam path.

## Abstract 2

A narrow microbeam is shown to be more effective than a wide microbeam for microbeam radiation therapy.

## 1. Introduction

Microbeam radiation therapy (MRT), which was originally introduced for the treatment of brain tumors by Slatkin *et al.* (1992 ▶), uses a parallel array of microbeams, the so-called ‘co-planar microbeam’, composed of high-intensity and highly directional X-rays generated at a synchrotron radiation facility. The principle of this treatment is based on the high resistance of normal brain tissue to such irradiation. This phenomenon was first observed in experiments concerning the biological effects of cosmic rays in the late 1950s. Zeman *et al.* (1961 ▶) reported that a microscopic (25 µm) 22 MeV deuteron beam required a dose of over 4000 Gy to kill cells in the beam path in the mouse cortex, compared with a macroscopic (1 mm) beam of only 140 Gy which destroyed all tissue in its path.

Recently, Bräuer-Krisch *et al.* (2010 ▶) comprehensively reviewed the several *in vivo* studies of MRT that have been carried out in rodents. These studies used various tumor cell lines: glioma (Schültke *et al.*, 2008 ▶), gliosarcoma (Laissue *et al.*, 1998 ▶; Dilmanian *et al.*, 2002 ▶; Smilowitz *et al.*, 2006 ▶; Regnard *et al.*, 2008 ▶; Serduc *et al.*, 2008 ▶, 2009*a* ▶,*b* ▶), squamous cell carcinoma (Miura *et al.*, 2006 ▶) and mammary tumor (Dilmanian *et al.*, 2003 ▶) cell lines. The implantation site was either brain parenchyma or the flanks near the hind legs. The results of these studies provided clear evidence that MRT was associated with the suppression of tumor growth (Dilmanian *et al.*, 2003 ▶; Miura *et al.*, 2006 ▶) and the extension of life of the rodents implanted with tumors (Laissue *et al.*, 1998 ▶; Dilmanian *et al.*, 2002 ▶; Smilowitz *et al.*, 2006 ▶, 2008 ▶, 2009*a* ▶,*b* ▶; Regnard *et al.*, 2008 ▶; Schültke *et al.*, 2008 ▶).

**Figure 1:**
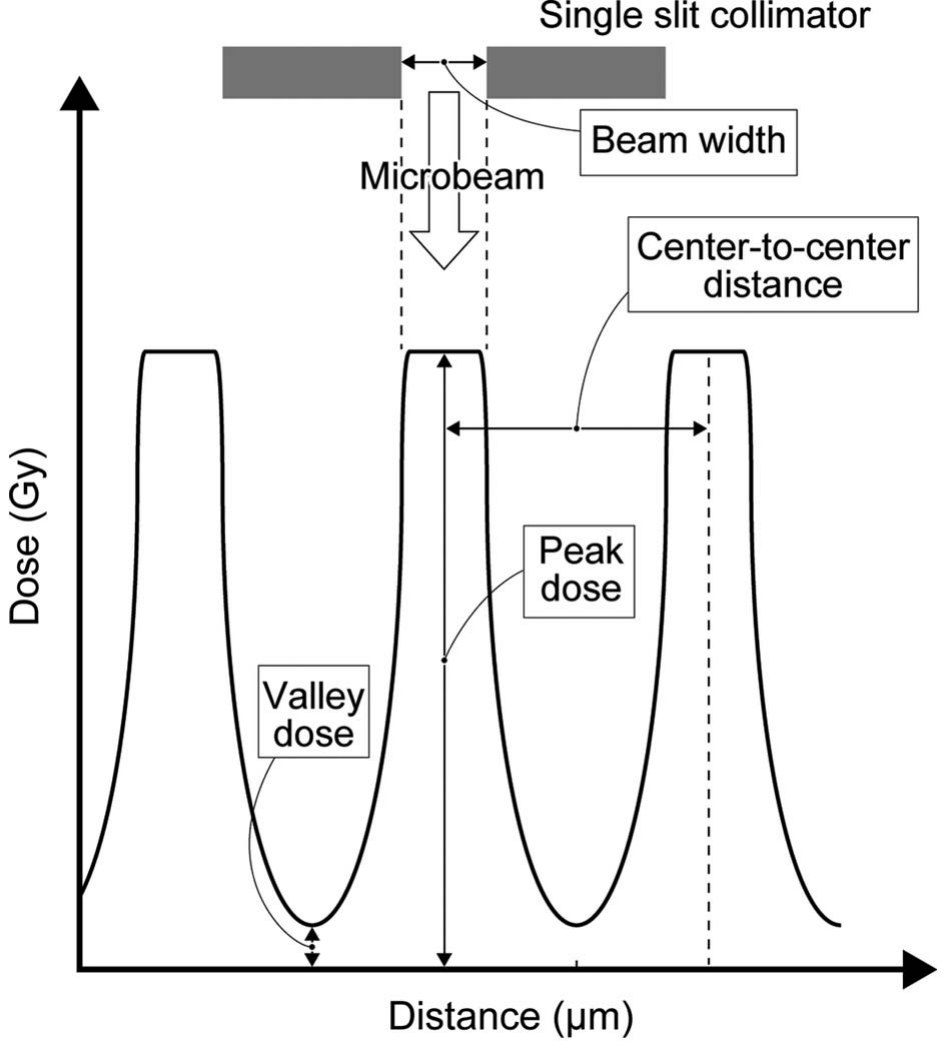
Schematic geometry of microbeam arrays used in MRT.

In MRT, the geometry of the microbeam is defined by the parameters of beam width, center-to-center distance, peak dose and valley dose (Fig. 1 ▶). In previous reports these parameters were in the range 25–90 µm, 50–300 µm, 150–900 Gy and 12.1–40 Gy, respectively (Laissue *et al.*, 1998 ▶; Dilmanian *et al.*, 2002 ▶, 2003 ▶; Miura *et al.*, 2006 ▶; Smilowitz *et al.*, 2006 ▶; Schültke *et al.*, 2008 ▶; Regnard *et al.*, 2008 ▶; Serduc *et al.*, 2008 ▶, 2009*a* ▶,*b* ▶). Most of these MRT studies used a narrow beam with a width of around 30 µm. However, such a narrow beam can be generated only by a large-scale synchrotron radiation facility. Since the number of such facilities is limited, a more practical beam for clinical purposes, the so-called ‘minibeam’ or ‘thick microbeam’, has recently been introduced for radiation therapy (Dilmanian *et al.*, 2006 ▶, 2008 ▶; Anschel *et al.*, 2007 ▶; Prezado *et al.*, 2009 ▶). A parallel array of thick beams (500–700 µm) is used to produce such a beam. However, the tumoricidal effects obtained with different beam widths have not yet been compared. It should also be emphasized that previous studies have needed a high valley dose (12.1–40 Gy), which might result in unacceptable irradiation levels in the valley area. Indeed, Bräuer-Krisch *et al.* (2010 ▶) state that the valley dose is the most important determinant of normal tissue damage in MRT.

The purpose of this study was to compare tumor growth in human U251 glioma cells following microbeam radiation treatment using microbeams of two different widths (20 µm and 100 µm). For this purpose an adjustable collimator, which enables modulation of the variable peak width, was used for the first time. To avoid any tumoricidal effect caused by valley irradiation, we chose a relatively low dose of irradiation, with the valley dose set as low as 4.8–9.6 Gy. We then assessed the effect of MRT by measuring the volume of tumors irradiated over time and recording the histological findings of tumors in the acute phase after irradiation.

## 2. Materials and methods

### 2.1. Experimental groups

Thirty-six mice implanted with tumors were divided into a MRT-treated group (*n* = 28) and a control group (*n* = 8). The MRT-treated group was then further divided into four subgroups: (i) a co-planar MRT group where microbeams 100 µm wide with a 500 µm center-to-center distance were used (‘co-planar 100’; *n* = 8); (ii) a cross-planar MRT group where microbeams 100 µm wide with a 500 µm center-to-center distance were used (‘cross-planar 100’; *n* = 8); (iii) a cross-planar MRT group where microbeams 20 µm wide with a 100 µm center-to-center distance were used (‘cross-planar 20’; *n* = 6); and (iv) a repeated cross-planar MRT group where microbeams 100 µm wide with a 500 µm center-to-center distance were delivered once a day for two days (‘cross-planar 100 × 2’; *n* = 6).

### 2.2. Preparation of tumor model

All procedures involving animals were approved by the Animal Care and Use Review Committee of Kobe University Graduate School of Medicine. Male five-week-old nude mice (BALB/cAJc1-nu/nu) weighing 20–25 g (Clea Japan, Osaka, Japan) were housed in an approved specific pathogen-free facility at Kobe University in accordance with Laboratory Animal Resources Commission standards. Appropriate care was taken to minimize animal discomfort, and appropriate sterile surgical techniques were utilized for tumor implantation and drug administration. U251 human glioma cells were maintained in Dullbecco’s modified Eagle’s medium containing glutamine, 10% fetal bovine serum, penicillin/streptomycin, and grown at 310 K in a 5% CO2 incubator. Ten days before MRT, the tumor cells were concentrated to 6 × 10^6^ per 200 µl and implanted subcutaneously into the flanks near the hind legs of mice anesthetized with halothane inhalation.

### 2.3. Radiation source

MRT was performed at SPring-8, a large-scale synchrotron radiation facility in Japan. The radiation source was generated at the white X-ray bending-magnet beamline BL28B2. The radiation beam traveled in a vacuum transport tube with minimized air scattering of the primary beam. X-rays passed from the vacuum tube into the atmosphere through a beryllium vacuum window, then into a 2.0 m helium beam path consisting of an aluminium tube and a thin aluminium helium window located 42 m from the synchrotron radiation output. The sample positioning system was placed 2.5 m from the thin aluminium window. A 3 mm-thick copper filter was inserted into the beam to remove the low-energy component. The X-ray spectrum was in the range 50–200 keV, peaking at around 90 keV. The air kerma rate of the broad beam was measured with a free-air ionization chamber. The electrode gap was 85 mm, which kept the electron escape fraction from the chamber below 3%, at 50–200 keV (Nariyama *et al.*, 2004 ▶). Near current saturation was obtained by applying a voltage of 9.5 kV.

### 2.4. Collimator and irradiation

**Figure 2:**
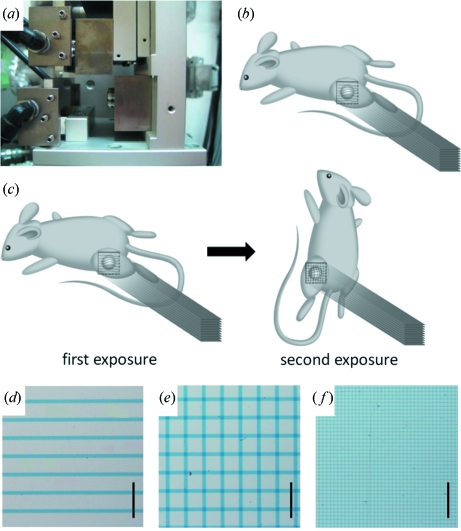
(*a*) An adjustable single-slit collimator which enables modulation of the variable peak width. (*b*) For co-planar microbeam irradiation, mice were treated with a single set of irradiation in the prone position. (*c*) For cross-planar microbeam irradiation, mice were irradiated in stages by means of 90° rotation about the axis parallel to the microbeams. (*d*–*f*) The spatial dose distribution was confirmed using GafChromic film for ‘co-planar 100’ (*d*), ‘cross-planar 100’ (*e*) and ‘cross-planar 20’ (*f*). Scale bar: 1 mm.

MRT was performed with the aid of an adjustable single-slit collimator which enabled a variable spatial fractionation of the X-ray beam (Fig. 2*a* ▶). The microbeam width was equal to the distance between two plates of tantalum. The center-to-center distance between one beam and the next beam was determined by horizontally moving the platform holding the experimental animal. An anesthetized mouse (sodium pentobarbital; 0.5 mg per 10 g of body weight, i.p.) was placed on the platform in the prone position lying on a styrol box. The hind leg with the tumor was immobilized with a plastic ring in a direction perpendicular to the microbeams. For the ‘co-planar MRT’ irradiation, mice received a single irradiation treatment in the prone position (Fig. 2*b* ▶); for the ‘cross-planar MRT’, mice received staged irradiation, first receiving irradiation as performed for ‘co-planar MRT’, followed by a second irradiation in the vertical position by rotating the axis by 90° so that the head was up (Fig. 2*c* ▶). The radiation field was 15 mm wide and 15 mm high.

The spatial dose distribution was examined using GafChromic film HD-810 (ISP Technologies, NJ, USA) as described previously (Nariyama *et al.*, 2009 ▶) (Figs. 2*d*–2*f* ▶). The optical density of the irradiated films was measured with a digital microscope through bandpass filters, and converted to dose using a calibration curve obtained in advance. The bandpass filters of 601 and 668 nm were used to attain the straight line for the calibration curve and increase the sensitivity and accuracy; the former was used for the peak dose and the latter for the valley dose.

### 2.5. Dose rate setting

**Figure 3:**
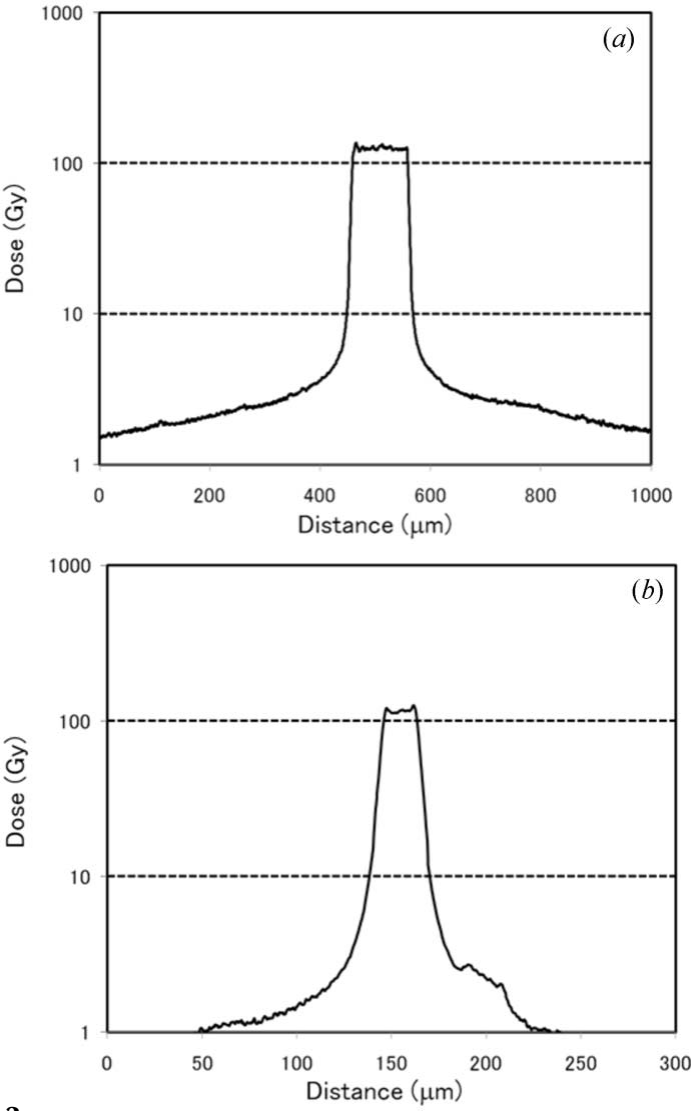
The spatial dose distribution was measured using GafChromic film in a 100 µm-wide microbeam (*a*) and 20 µm-wide microbeam (*b*). The peak dose was 130 Gy for both.

The air kerma rate was preset at 140 Gy s^−1^ at the hutch. With the newly designed adjustable collimeter the X-ray peak dose rates were found to be 124 Gy s^−1^ and 111 Gy s^−1^ for the 100 µm and 20 µm microbeams, respectively (Figs. 3*a* and 3*b* ▶). The dose rate at a distance of 250 µm from the center of the 100 µm microbeam was 4.8 Gy s^−1^ (Fig. 3*a* ▶), while that at a distance of 50 µm from the center of the 20 µm microbeam was 4.1 Gy s^−1^ (Fig. 3*b* ▶). The duration of irradiation was 1 s, in order to keep the valley dose low while ensuring sufficient cell damage at peak dose. The valley dose of the cross-planar microbeam was double that of the co-planar microbeam at the same peak dose. For ‘co-planar 100’, ‘cross-planar 100’ and ‘cross-planar 20’, the cumulative valley dose was 4.8 Gy, 9.6 Gy and 8.2 Gy, respectively.

### 2.6. Evaluation of tumor growth

Tumor volume was measured for the two perpendicular diameters (*X*, *Y*) and thickness (*Z*) two to three times per week for one month after irradiation, by a technical assistant who was not informed of the treatment protocol. Tumor volume (*V*) was estimated using the formula *V* = *X* × *Y* × *Z* × 0.52 as described previously (Shichiri *et al.*, 2009 ▶). The relative growth ratio was defined as *V*(at individual measurement point)/*V*(at irradiation) and analyzed statistically.

### 2.7. Histopathology

Forty-two mice were used for histopathological analysis. To determine the response to MRT, mice from the ‘co-planar 100’, ‘cross-planar 100’ and ‘cross-planar 20’ groups (excluding the ‘cross-planar 100 × 2’ group) were sacrificed at 24 and 72 h after irradiation (for all groups *n* = 3 for each time point). Three tumor-bearing mice that did not receive irradiation were sacrificed as controls at each of the same time points. To examine rapid pathological changes in the early post-irradiation phase, additional mice in the ‘cross-planar 100’ and ‘cross-planar 20’ groups were sacrificed at 3, 6 and 12 h after irradiation (*n* = 3 for each time point). After the mice had been deeply anesthetized, tumors were removed and fixed with 4% paraformaldehyde in phosphate-buffered saline (pH 7.4) for 24 h. Tumor tissue was cut along the horizontal plane perpendicular to the microbeams, embedded in paraffin, processed to yield 4 µm-thick sections, and stained with hematoxylin and eosin (HE).

Sections from the ‘cross-planar 100’ and ‘cross-planar 20’ groups obtained 24 and 72 h after irradiation as well as sections from the control mice were also used for detecting apoptosis by using the terminal deoxynucleotidyl transferase-mediated dUTP nick-end labeling (TUNEL) technique. The TUNEL reaction was performed with the ApoMark DNA Fragmentation Apoptosis Detection Kit (Exalpha Biologicals, Shirley, MA, USA) according to the manufacturer’s instructions. The rate of apoptosis, calculated as the percentage of TUNEL-positive cells out of 1000 cells, was determined for 12 random tumor sections taken from three different tumors in each group.

### 2.8. Statistics

A one-way ANOVA was used to examine within-group differences in the tumor growth ratio at the individual time points post-irradiation and the difference in percentage of TUNEL-positive cells. For comparison between the groups, an additional *post-hoc* test was performed using the Turkey–Kramer method. The threshold for statistical significance was set at *p* < 0.05. Values are expressed in the figures as mean ± standard deviation (SD).

## 3. Results

### 3.1. Tumor growth

**Figure 4:**
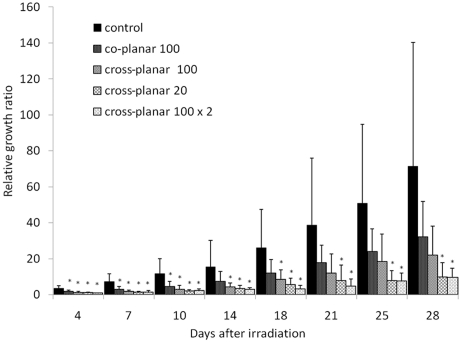
The relative growth ratios at various measurement time points during a 28 day period compared with measurements obtained at irradiation for ‘control’, ‘co-planar 100’, ‘cross-planar 100’, ‘cross-planar 20’ and ‘cross-planar 100 × 2’. The asterisks indicate *p* < 0.05 when compared with the control group at a given time point.

At irradiation the mean tumor volume (mm^3^) for the control, ‘co-planar 100’, ‘cross-planar 100’, ‘cross-planar 20’ and ‘cross-planar 100 × 2’ groups were 43.8 ± 17.1, 37.1 ± 14.5, 34.8 ± 10.6, 34.7 ± 7.1 and 40.6 ± 13.3 (mean ± SD), respectively. As shown in Fig. 4 ▶, the groups showed suppression from lowest to highest in the order of ‘co-planar 100’, ‘cross-planar 100’, ‘cross-planar 20’ and ‘cross-planar 100 × 2’. At 28 days after irradiation, tumor growth ratios of the control, ‘co-planar 100’, ‘cross-planar 100’, ‘cross-planar 20’ and ‘cross-planar 100 × 2’ groups reached 71.5 ± 68.7, 32.2 ± 19.6, 22.0 ± 16.2, 9.9 ± 7.9 and 9.6 ± 5.2, respectively (Fig. 4 ▶). When compared with the control group, the ‘co-planar 100’ group showed significant suppression of tumor growth (*p* < 0.05) at 4, 7 and 10 days post-irradiation, the ‘cross-planar 100’ group showed significant suppression at 4, 7, 10, 14 and 18 days post-irradiation, and the ‘cross-planar 20’ and ‘cross-planar 100 × 2’ showed significant suppression at 4, 7, 10, 14, 18, 21, 25 and 28 days post-irradiation. Thus, the ‘cross-planar 20’ group showed longer tumor growth suppression than the ‘cross-planar 100’ group, and the ‘cross-planar 100 × 2’ group showed suppression comparable with that of the ‘cross-planar 20’ group.

### 3.2. Histological study

**Figure 5:**
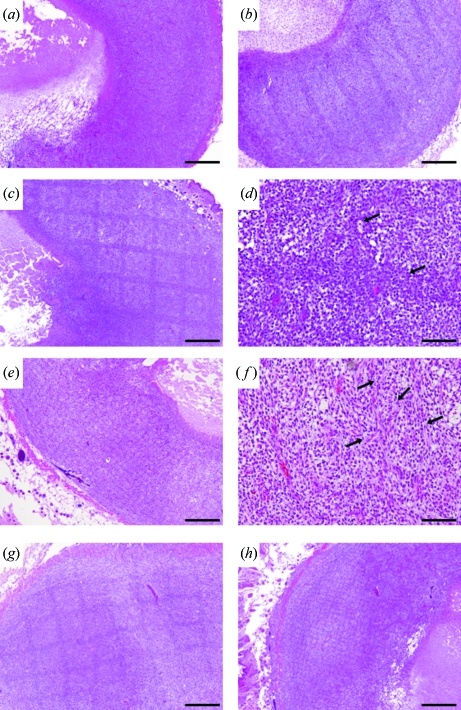
HE-stained sections of control (*a*), and 24 h after irradiation with ‘co-planar 100’ (*b*), ‘cross-planar 100’ (*c*–*d*) and ‘cross-planar 20’ (*e*–*f*), showing dark stripes along the beam path. The dark stripes remained 72 h after irradiation with ‘cross-planar 100’ (*g*) and ‘cross-planar 20’ (*h*). Arrows: microbeam path. Scale bar for (*a*), (*b*), (*c*), (*e*), (*g*), (*h*): 500 µm; for (*d*), (*f*): 100 µm.

**Figure 6:**
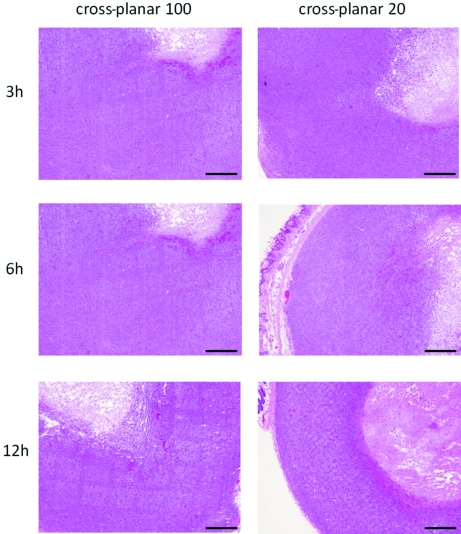
HE-stained sections 3, 6 and 12 h after irradiation with ‘cross-planar 100’ and ‘cross-planar 20’. Scale bar: 500 µm.

HE-stained sections of the non-irradiated control mice showed a large and dense cellular mass with marked pleomorphism. Endothelial hypertrophy was not evident, a few necrotic regions were located in the center of mass, and typical palisading cells were seen around the necrotic lesion (Fig. 5*a* ▶). Irradiated HE-stained sections showed dark stripes along the beam path at low magnification at 24 h post-irradiation. The path was clearly seen in the ‘co-planar 100’, ‘cross-planar 100’ and ‘cross-planar 20’ groups (Figs. 5*b*, 5*c*, 5*e* ▶), and a dense closely compacted cellular arrangement was observed on the path (Figs. 5*d*, 5*f* ▶). The nuclei were more darkly stained than the nuclei of non-irradiated tumors. There were no microhemorrhages or non-viable cells in the area between the peaks. The path itself in the ‘cross-planar 20’ group was narrow and faint, and the area between the peaks showed intercellular edema. The size of the necrotic lesion differed in each of the treated tumors but no small cavitations that would indicate new pathological developments were seen (Figs. 5*d*, 5*f* ▶). The dark stripes along the beam path remained at 72 h post-irradiation (Figs. 5*g*, 5*h* ▶). A comparison of the rapid pathological changes in the early post-irradiation phase in the ‘cross-planar 100’ and ‘cross-planar 20’ groups revealed darkly stained nuclei on the beam path in both groups at 6 h post-irradiation (Fig. 6 ▶).

**Figure 7:**
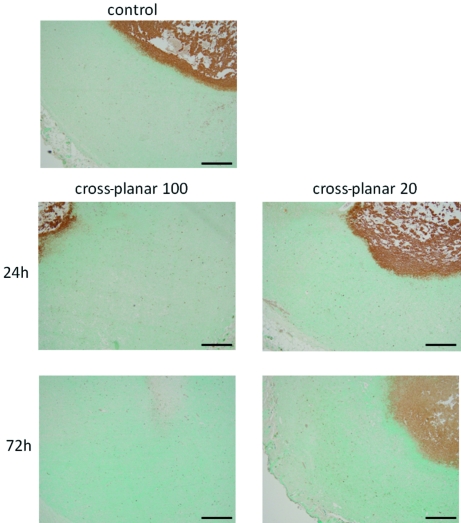
TUNEL staining of the sections irradiated with ‘cross-planar 100’ and ‘cross-planar 20’ showing few apoptotic regions in any of the irradiated fields at low magnification. Scale bar: 500 µm.

**Figure 8:**
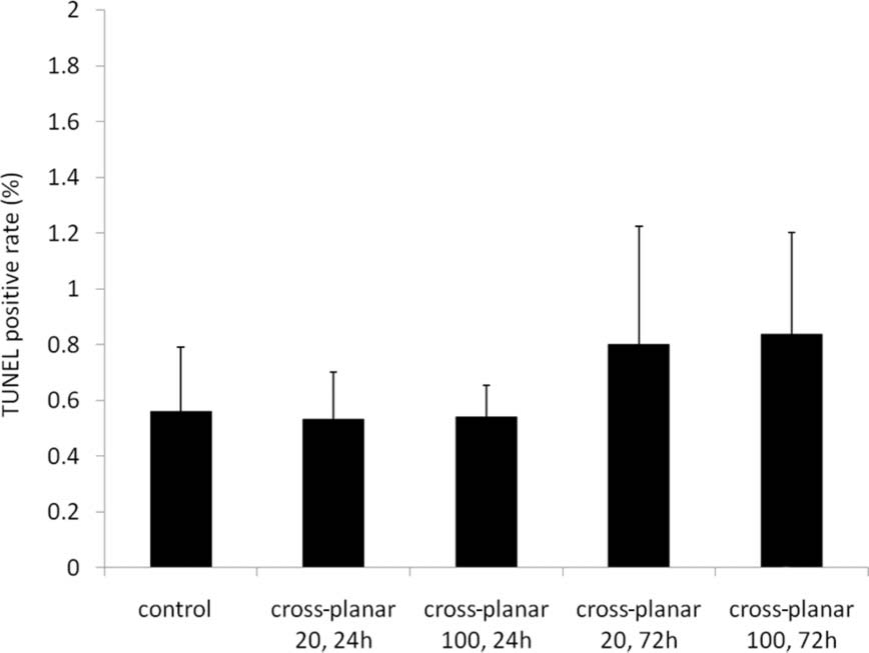
The percentages of TUNEL-positive cells are summarized.

TUNEL results showed few apoptotic regions in all of the irradiated fields (Fig. 7 ▶). The average percentage of TUNEL-positive cells was 0.56 ± 0.23 in the control group and 0.54 ± 0.11 in the ‘cross-planar 100’ group at 24 h post-irradiation, 0.53 ± 0.17 in the ‘cross-planar 100’ group at 72 h, 0.84 ± 0.37 in the ‘cross-planar 20’ group at 24 h, and 0.80 ± 0.42 in the ‘cross-planar 100’ group at 72 h (Fig. 8 ▶). The percentage was not significantly different between any two groups.

## 4. Discussion

We studied the effects of MRT with a greater beam width and center-to-center distance than reported previously in an animal model implanted with U251 human glioma cells. We demonstrated that microbeams with a greater beam width and center-to-center distance (100 µm and 500 µm, respectively) than reported previously (25–90 µm and 50–300 µm, respectively) produced moderate tumor growth suppression when applied in a cross-planar pattern, and that narrow microbeams with a width of 20 µm showed longer tumor growth suppression than microbeams with a width of 100 µm. These findings indicate that the tumor suppression effect of X-ray irradiation does not depend on the total amount of irradiated dose alone. Differences in spatial distribution also clearly affect tumor growth suppression and a narrow beam is more effective than a wide beam for MRT.

With regard to the mechanisms of tumor growth suppression, it could be suggested that the bystander effect of MRT affects tumor cells in the valley zone. However, our previous *in vitro* study using C6 glioma cells (Kashino *et al.*, 2009 ▶) demonstrated that such an effect is not sufficient to explain *in vivo* tumor growth suppression. At least, MRT-treated cells cultured in a dish do not exactly mimic the characteristics of MRT-treated cells *in vivo*.

Another possibility is that the tumoricidal effect of MRT results from the high biologically hazardous dosing of the valley zone, in other words, the background irradiation of all the targeted areas. To clarify this point we reviewed all previous MRT studies using animals reported in the literature (Table 1 ▶). Regardless of differences in tumor cell line, irradiation geometry, beam width, center-to-center distance or peak dose, we found that the valley doses used in these studies were relatively high (12.1–40 Gy) and this has a direct effect on tumor cell growth in the valley zone. In particular, this dose could be critical when radiosensitive tumor cells are used. In previous studies of experimental radiosurgery on malignant brain tumors using a gamma knife unit the 50% marginal dose was 15–35 Gy, and cellular damage was histologically proven and survival rate significantly improved (Kondziolka *et al.*, 1992 ▶; Niranjan *et al.*, 2000 ▶, 2003 ▶; Nakahara *et al.*, 2001 ▶). To minimize the direct effect in the valley zone, our study used a lower dose (4.8–9.6 Gy) than used previously. Since a radiation dose <10 Gy has never been reported to affect tumor cells *in vivo*, tumor growth suppression in the present study is unlikely to have resulted from the valley dose.

A double-strand break of DNA is fundamentally a direct acute cellular response to radiation and probably occurs in the peak area of both the wider (100 µm) and narrower (20 µm) microbeam. One unexpected result of the present study was that no significant change in the rate of apoptotic cells was detected by TUNEL staining. In another study it was found that a 35–70 Gy dose administered with a gamma knife unit did induce apoptotic cell death of 9L gliosarcoma between 6 and 48 h post-irradiation (Witham *et al.*, 2005 ▶) in a treated area 4 mm in diameter. However, in our study, microbeams of the order of 20 to 100 µm did not result in apoptotic tumor cell death even in the peak zone. No tissue death was induced either at the cellular or tissue level, because the necrotic area in each subgroup hardly changed after MRT. Therefore, the mechanism of tumor growth suppression in our study is likely to be the induction of lower cell proliferation.

Another possible mechanism of *in vivo* tumor suppression may be alteration of microvascular structures. Serduc *et al.* (2008 ▶) hypothesized that the tumoral vessel injury caused by MRT mainly affected tumor growth suppression, but they found no significant microvascular components, at least under their experimental conditions using 9L gliosarcoma implanted into the brain. We believe that further histological or functional studies of neovascularizing tumor vessels are required to identify the tumoricidal mechanism of MRT.

Recently, radiation therapy using a parallel array of thick beams (500–700 µm) with the same separation distance between beams, the so-called ‘minibeam’ or ‘thick microbeam’ therapy, has been recommended (Dilmanian *et al.*, 2006 ▶, 2008 ▶; Anschel *et al.*, 2007 ▶; Prezado *et al.*, 2009 ▶). Dilmanian *et al.* (2008 ▶) reported that this irradiation method at a peak dose of 170 Gy did not induce neurological deficits in rats. Although the biological mechanisms of the effects of such minibeams on tumor cells have not been thoroughly studied, our study demonstrated that the wide MRT of ‘cross-planar 100’ was as effective as the narrow MRT of ‘cross-planar 20’ when the wide MRT was applied once a day for two days. We therefore think that temporal fractionated MRT is useful for amplification of the tumoricidal effect of MRT.

Maintaining the proper balance between the tumoricidal and adverse effects of MRT on normal brain tissue and function is a challenging aspect in the refinement of the therapy. Regnard *et al.* (2008 ▶) reported on the effects of MRT by comparing the results obtained with 25 µm-wide co-planar beams with a 200 µm or 100 µm center-to-center distance. They found that MRT with a 200 µm center-to-center distance was superior in terms of sparing healthy tissue but that lifespan was longer with a 100 µm center-to-center distance. Schültke *et al.* (2008 ▶) reported that no memory dysfunction was detected in object recognition tests for rats treated with brain irradiation using 25 µm-wide microbeams with a 200 µm center-to-center distance and a skin entrance dose of 350 Gy. Whether wide MRT of 100 µm may affect normal brain tissue or function remains to be determined in future research.

In conclusion, MRT using a 100 µm-wide microbeam with 500 µm center-to-center distance resulted in moderate tumor growth suppression, although MRT using a 20 µm-wide microbeam resulted in longer tumor growth suppression. The biological mechanism underlying these findings is still unclear: it may involve functional tissue deterioration rather than direct cellular damage in the beam path. Further comparative experimental studies using both wide and narrow microbeams are warranted to determine the potential of MRT for clinical purposes.

## Figures and Tables

**Table 1:** Summary of previously reported experiments of MRT with animals

Author; journal; year	Tumor cell line	Implantation site	Irradiation geometry	Beam width (µm)	Center-to-center distance (µm)	Peak dose (Gy)	Valley dose (Gy)	Evaluation criteria
Laissue *et al.*; *Int. J. Cancer*; 1998	9L gliosarcoma	Brain	Co-planar	25	100	625		Survival rate
			Orthogonal	25	100	312.5		Tumor size
			Orthogonal	25	100	625		Normal brain damage
Dilmanian *et al.*; *Neuro-Oncology*; 2002	9L gliosarcoma	Brain	Co-planar	27	50	150–300	20–40	Survival rate
			Co-planar	27	75	250–500	17–33	MRI (tumor size, normal brain damage)
			Co-planar	27	100	500	19	
Dilmanian *et al.*; *Radiat. Res.*; 2003	Murine EMT-6 mammary carcinoma	Hind leg	Co-planar	90	300	800–1900	16–38	Tumor size
			Cross-planar	90	300	410–650	16–26	Normal tissue toxicity
Smilowitz *et al.*; *J. Neuro-oncology*; 2006	9L gliosarcoma	Brain	Co-planar	25	211	625		Survival rate
Miura *et al.*; *Br. J. Radiol.*; 2006	Human squamaous cell carcinoma	Hind leg	Orthogonal	35	200	442		Tumor size
			Orthogonal	35	200	625		Normal tissue toxicity
			Orthogonal	35	200	884		
			Orthogonal	70	200	442		
Regnard *et al.*; *Phys. Med. Biol.*; 2008	9L gliosarcoma	Brain	Co-planar	25	100	625	36	Survival rate
			Co-planar	25	200	625	12.1	Clinical sign
								Body weight pattern
Serduc *et al.*; *Phys. Med. Biol.*; 2008	9L gliosarcoma	Brain	Orthogonal	25	211	500	24	Survival rate
								MRI (blood volume, vessel size)
Schültke; *Eur. J. Radiol.*; 2008	F98 glioma	Brain	Orthogonal	25	211	350		Survival rate
	C6 glioma							
Serduc *et al.*; *Phys. Med. Biol.*; 2009	9L gliosarcoma	Brain	Orthogonal	25	211	860	36	Survival rate
			Orthogonal	50	211	480	36	
			Orthogonal	75	211	320	36	
Serduc *et al.*; *J. Synchrotron Rad.*; 2009	9L gliosarcoma	Brain	Three fractions through three orthogonal ports at 24 h intervals	50	211	400 (two directions)	15 per exposure	Survival rate
						360 (one direction)		Memory function
This work	Human U251 glioma	Hind leg	Co-planar	100	500	124	4.8	Tumor size
			Cross-planar	100	500	124	9.6	
			Cross-planar	20	100	111	8.2	

## References

[bb1] Anschel, D. J., Romanelli, P., Benveniste, H., Foerster, B., Kalef-Ezra, J., Zhong, Z. & Dilmanian, F. A. (2007). *Minim. Invas. Neurosurg.* **50**, 43–46. 10.1055/s-2007-976514 17546543

[bb2] Bräuer-Krisch, E., Serduc, R., Siegbahn, E. A., Le Duc, G., Prezado, Y., Bravin, A., Blattmann, H. & Laissue, J. A. (2010). *Mutat. Res.* **704**, 160–166. 10.1016/j.mrrev.2009.12.003 20034592

[bb3] Dilmanian, F. A., Button, T. M., Le Duc, G., Zhong, N., Pena, L. A., Smith, J. A., Martinez, S. R., Bacarian, T., Tammam, J., Ren, B., Farmer, P. M., Kalef-Ezra, J., Micca, P. L., Nawrocky, M. M., Niederer, J. A., Recksiek, F. P., Fuchs, A. & Rosen, E. M. (2002). *Neuro-Oncology*, **4**, 26–38. 10.1215/15228517-4-1-26 PMC1920629 11772430

[bb4] Dilmanian, F. A., Morris, G. M., Zhong, N., Bacarian, T., Hainfeld, J. F., Kalef-Ezra, J., Brewington, L. J., Tammam, J. & Rosen, E. M. (2003). *Radiat. Res.* **159**, 632–641. 10.1667/0033-7587(2003)159[0632:mechte]2.0.co;2 12710874

[bb5] Dilmanian, F. A., Romanelli, P., Zhong, Z., Wang, R., Wagshul, M. E., Kalef-Ezra, J., Maryanski, M. J., Rosen, E. M. & Anschel, D. J. (2008). *Eur. J. Radiol.* **68**, S129–S136. 10.1016/j.ejrad.2008.04.055 18606516

[bb6] Dilmanian, F. A., Zhong, Z., Bacarian, T., Benveniste, H., Romanelli, P., Wang, R., Welwart, J., Yuasa, T., Rosen, E. M. & Anschel, D. J. (2006). *Proc. Natl Acad. Sci. USA*, **103**, 9709–9714. 10.1073/pnas.0603567103 PMC1480471 16760251

[bb7] Kashino, G., Kondoh, T., Nariyama, N., Umetani, K., Ohigashi, T., Shinohara, K., Kurihara, A., Fukumoto, M., Tanaka, H., Maruhashi, A., Suzuki, M., Kinashi, Y., Liu, Y., Masunaga, S., Watanabe, M. & Ono, K. (2009). *Int. J. Radiat. Oncol. Biol. Phys.* **74**, 229–236. 10.1016/j.ijrobp.2008.09.060 19362241

[bb8] Kondziolka, D., Lunsford, L. D., Claassen, D., Pandalai, S., Maitz, A. H. & Flickinger, J. C. (1992). *Neurosurgery*, **31**, 280–287. 10.1227/00006123-199208000-00013 1325039

[bb9] Laissue, J. A., Geiser, G., Spanne, P. O., Dilmanian, F. A., Gebbers, J. O., Geiser, M., Wu, X. Y., Makar, M. S., Micca, P. L., Nawrocky, M. M., Joel, D. D. & Slatkin, D. N. (1998). *Int. J. Cancer*, **78**, 654–660. 10.1002/(sici)1097-0215(19981123)78:5<654::aid-ijc21>3.0.co;2-l 9808538

[bb10] Miura, M., Blattmann, H., Bräuer-Krisch, E., Bravin, A., Hanson, A. L., Nawrocky, M. M., Micca, P. L., Slatkin, D. N. & Laissue, J. A. (2006). *Br. J. Radiol.* **79**, 71–75. 10.1259/bjr/50464795 16421408

[bb11] Nakahara, N., Okada, H., Witham, T. F., Attanucci, J., Fellows, W. K., Chambers, W. H., Niranjan, A., Kondziolka, D. & Pollack, I. F. (2001). *J. Neurosurg.* **95**, 984–989. 10.3171/jns.2001.95.6.0984 11765844

[bb12] Nariyama, N., Kishi, N. & Ohnishi, S. (2004). *Nucl. Instrum. Methods Phys. Res. A*, **524**, 324–331.

[bb13] Nariyama, N., Ohigashi, T., Umetani, K., Shinohara, K., Tanaka, H., Maruhashi, A., Kashino, G., Kurihara, A., Kondob, T., Fukumoto, M. & Ono, K. (2009). *Appl. Radiat. Isot.* **67**, 155–159. 10.1016/j.apradiso.2008.08.002 18789708

[bb14] Niranjan, A., Moriuchi, S., Lunsford, L. D., Kondziolka, D., Flickinger, J. C., Fellows, W., Rajendiran, S., Tamura, M., Cohen, J. B. & Glorioso, J. C. (2000). *Mol. Ther.* **2**, 114–120. 10.1006/mthe.2000.0101 10947938

[bb15] Niranjan, A., Wolfe, D., Tamura, M., Soares, M. K., Krisky, D. M., Lunsford, L. D., Li, S., Fellows-Mayle, W., DeLuca, N. A., Cohen, J. B. & Glorioso, J. C. (2003). *Mol. Ther.* **8**, 530–542. 10.1016/s1525-0016(03)00232-6 14529825

[bb16] Prezado, Y., Renier, M. & Bravin, A. (2009). *J. Synchrotron Rad.* **16**, 582–586. 10.1107/S0909049509012503 19535874

[bb17] Regnard, P., Le Duc, G., Bräuer-Krisch, E., Troprès, I., Siegbahn, E. A., Kusak, A., Clair, C., Bernard, H., Dallery, D., Laissue, J. A. & Bravin, A. (2008). *Phys. Med. Biol.* **53**, 861–878. 10.1088/0031-9155/53/4/003 18263945

[bb18] Schültke, E., Juurlink, B. H., Ataelmannan, K., Laissue, J., Blattmann, H., Bräuer-Krisch, E., Bravin, A., Minczewska, J., Crosbie, J., Taherian, H., Frangou, E., Wysokinsky, T., Chapman, L. D., Griebel, R. & Fourney, D. (2008). *Eur. J. Radiol.* **68**, S142–S146. 10.1016/j.ejrad.2008.04.051 18614312

[bb19] Serduc, R., Bouchet, A., Bräuer-Krisch, E., Laissue, J. A., Spiga, J., Sarun, S., Bravin, A., Fonta, C., Renaud, L., Boutonnat, J., Siegbahn, E. A., Esteve, F. & Le Duc, G. (2009*a*). *Phys. Med. Biol.* **54**, 6711–6724. 10.1088/0031-9155/54/21/017 19841517

[bb20] Serduc, R., Bräuer-Krisch, E., Bouchet, A., Renaud, L., Brochard, T., Bravin, A., Laissue, J. & Le Duc, G. (2009*b*). *J. Synchrotron Rad.* **16**, 587–590. 10.1107/S0909049509012485 19535875

[bb21] Serduc, R., Christen, T., Laissue, J., Farion, R., Bouchet, A., Sanden, B., Segebarth, C., Bräuer-Krisch, E., Le Duc, G., Bravin, A., Rémy, C. & Barbier, E. L. (2008). *Phys. Med. Biol.* **53**, 3609–3622. 10.1088/0031-9155/53/13/015 18560052

[bb22] Shichiri, M., Fukai, N., Kono, Y. & Tanaka, Y. (2009). *Cancer Res.* **69**, 4760–4768. 10.1158/0008-5472.CAN-08-3417 19458074

[bb23] Slatkin, D. N., Spanne, P., Dilmanian, F. A. & Sandborg, M. (1992). *Med. Phys.* **19**, 1395–1400. 10.1118/1.596771 1461201

[bb24] Smilowitz, H. M., Blattmann, H., Bräuer-Krisch, E., Bravin, A., Di Michiel, M., Gebbers, J. O., Hanson, A. L., Lyubimova, N., Slatkin, D. N., Stepanek, J. & Laissue, J. A. (2006). *J. Neurooncol.* **78**, 135–143. 10.1007/s11060-005-9094-9 16598429

[bb25] Witham, T. F., Okada, H., Fellows, W., Hamilton, R. L., Flickinger, J. C., Chambers, W. H., Pollack, I. F., Watkins, S. C. & Kondziolka, D. (2005). *Stereotact. Funct. Neurosurg.* **83**, 17–24. 10.1159/000084475 15775705

[bb26] Zeman, W., Curtis, H. J. & Baker, C. P. (1961). *Radiat. Res.* **15**, 496–514. 14010109

